# Deep eutectic solvent assisted oil - water interfacial behavior on polystyrene surfaces: a computational study

**DOI:** 10.1038/s41598-025-19783-0

**Published:** 2025-10-15

**Authors:** Kalampyr Bexeitova, Aida Ardakkyzy, Raushan Soltan, Galiya Baisalova, Damen Nurgalieva, Mirat Karibayev, Zhexenbek Toktarbay

**Affiliations:** 1 Laboratory of Engineering Profile, Satbayev University, Satbayev St. 22a, Almaty, 050013 Kazakhstan; 2 L. N. Gumilyov Eurasian National University, 010000 Astana, Kazakhstan; 3 Astana Medical University, 010000 Astana, Kazakhstan; 4 Astana International University, 010000 Astana, Kazakhstan; 5 Laboratory of Renewable Energy, National Laboratory Astana, Nazarbayev University, 010000 Astana, Kazakhstan; 6 Institute of Natural Sciences, Nanotechnology and New Materials, Khoja Akhmet Yassawi International Kazakh-Turkish University, Bekzat Sattarhanov Street No 29, Turkestan, Kazakhstan

**Keywords:** Polystyrene, Membrane, Octane, Water, Deep eutectic solvents, Separation, Molecular dynamics, Chemistry, Engineering, Materials science

## Abstract

Oil-water separation on hydrophobic polymer surfaces, such as polystyrene (PS), faces significant challenges due to poor interfacial control and low wettability. This study computationally explores the efficacy of Deep Eutectic Solvents (DES) as environmentally friendly additives to modify interfacial behavior and enhance compatibility between oil, water, and the polymer surface. Utilizing classical all-atom molecular dynamics (MD) simulations, we investigated the molecular-level interactions and structural organization at the oil-water-DES-PS interface. Our molecular structural analysis reveals that DES preferentially adsorbs onto the PS surface, displacing water and facilitating enhanced contact between octane and PS. Radial Distribution Function (RDF) analysis quantifies this effect, showing a reduction in direct PS-water interactions and a strong, specific affinity between PS carbon atoms and DES choline. Furthermore, interaction energy analysis confirms that DES significantly strengthens the van der Waals attraction between PS and octane, primarily driven by the strong interaction of choline with PS. These molecular insights are crucial for the rational design of advanced, DES-assisted membrane systems, offering a promising avenue for efficient and sustainable oil-water separation.

## Introduction

Environmental pollution, particularly the contamination of water bodies caused by industrial discharge and domestic waste, has emerged as a pressing global issue. Among various pollutants, oily wastewater is of significant concern due to its widespread occurrence and detrimental impacts on both ecosystems and human health. This type of wastewater primarily originates from petrochemical industries, oil spills, food processing, transportation activities, and household discharges^1–5^. The persistent and toxic nature of oil in aquatic environments leads to reduced oxygen transfer, disrupted aquatic life, and long-term ecological imbalances. Therefore, the efficient and sustainable separation of oil from water has become a crucial task in global efforts toward water conservation and pollution mitigation.

The challenge of separating oil from water is compounded by the complex physicochemical nature of oil-water emulsions, which are often stabilized by surfactants, colloidal particles, and organic matter. These emulsions, particularly those with droplet sizes in the nano- and microscale exhibit high stability, making traditional separation techniques less effective^4–10^. Conventional methods for oil-water separation include gravity separation, coagulation-flocculation, air flotation, skimming, and chemical treatment. While effective under certain conditions, these approaches often suffer from limitations such as high energy demand, secondary pollution, low separation efficiency for stable emulsions, and reduced performance under varying pH or salinity levels.

In recent years, membrane-based technologies have emerged as a highly promising solution for oil-water separation due to their high selectivity, continuous operation potential, and scalability. These membranes act as physical barriers, allowing selective passage of water while retaining oil or vice versa, depending on their surface properties. Separation efficiency is primarily governed by membrane surface wettability, porosity, chemical compatibility, and mechanical strength^11–19^.

Various polymers have been explored as membrane materials, including cellulose derivatives, polyvinylidene fluoride (PVDF), polyethylene glycol diacrylate (PEGDA), and polysulfone (PSF). These materials have shown varying degrees of success in terms of separation performance, antifouling resistance, mechanical stability, and cost-effectiveness. Furthermore, advanced membrane engineering techniques such as blending, surface grafting, and nanoparticle incorporation have been employed to enhance membrane functionalities. In this context, surface modification using functional additives such as ionic liquids and deep eutectic solvents (DES) has gained considerable attention as a green and tunable approach to optimize membrane performance by altering interfacial characteristics^14–20^.

DES, composed of a hydrogen bond donor and hydrogen bond acceptor, offer low toxicity, high biodegradability, and tunable physicochemical properties^18–21^. These properties make DES promising candidates for enhancing membrane surface behavior, especially in challenging separation environments. Their ability to interact with both hydrophilic and hydrophobic phases makes them particularly suitable for modulating the oil-water interface, reducing interfacial tension, and improving the selectivity and permeability of membrane systems.

Among various membrane materials, polystyrene (PS) stands out due to its inherent hydrophobicity, ease of processing, thermal stability, and availability. PS-based membranes have shown potential in oil-water separation applications, especially when surface-modified to enhance selective wettability^20–25^. However, in their pristine form, PS membranes may lack sufficient interaction with dispersed water or oil phases, particularly in complex emulsions. To overcome these limitations, strategies involving the incorporation of DES can be employed to tune surface chemistry, improve interfacial compatibility, and potentially enhance separation performance.

Polymer-based membranes for oil-water separation have been widely studied, with distinct material systems offering unique benefits (Table 1). The table below summarizes relevant studies involving polymer membranes, including experimental and computational works.

**Table 1:** Recent work conducted for oil water separation.

Polymer type	Method	Key parameters	Conclusion
Cellulose Acetate (CA)/Nylon 66^24^	Solution casting with Dimethyl sulfoxide (DMSO) solvent	90% oil rejection, 33 L/m²h flux	Blending improves thermal/mechanical properties compared to pure CA.
PVDF)^24^	Modified with alumina nanoparticles	99% oil rejection, 1 mg/L oil in permeate	Nanoparticles enhance antifouling and separation efficiency.
PEGDA^24^	Hygro-responsive membrane design	> 99% efficiency, 210 L/m²h flux after 100 h	Superhydrophilic/superoleophobic properties maintain long-term performance.
PSF/CA^24^	Modified with zinc oxide nanoparticles	420 L/m²h flux	Modification improves hydrophilicity and antibacterial function.
Surface-modified PVDF^25^	Hydrophilic surface engineering	Reduced fouling, retained hydrophobicity	Hydrophilic coating enhances longevity and oil rejection.

From this comparison, it is evident that CA and PVDF dominate experimental studies due to their tunable hydrophilic properties and strong mechanical resilience. However, computational studies are shedding light on underexplored polymers such as polystyrene, offering atomistic insights into how interfacial behaviors can be tuned^24–28^. The current study focuses on computationally investigating the interaction of DES at oil-water-PS interfaces, with the aim of revealing molecular-level mechanisms that could guide the design of more efficient separation membranes.

To achieve this, we employ classical all-atom molecular dynamics (MD) simulations to examine the structural organization, dynamic behavior, and interfacial energetics of oil and water phases interacting with DES-modified PS surfaces. This method allows us to capture detailed insights into molecular interactions and surface wetting phenomena, thereby contributing valuable information to the rational design of advanced, DES-assisted membrane systems for oil-water separation.

In this work, we focus on understanding the molecular-level interactions between oil, water, and PS surfaces in the presence of DES, aiming to unlock new insights into interfacial behavior and separation performance. Using classical all-atom MD simulations, we investigate how DES influences the structuring, dynamics, and compatibility of oil and water at the PS interface.

## Materials and methods

### Computational models

In this study, classical all-atom MD simulations were carried out to investigate the effect of DES on oil–water interfacial behavior in the presence of PS surfaces. The primary aim was to elucidate how DES molecules interact at the interface and influence the structural and energetic characteristics of the system. A model system was constructed comprising three distinct components: a PS slab to represent the hydrophobic surface, a stratified oil–water interface, and varying amounts of DES molecules situated at or near the interface.

**Fig. 1:**
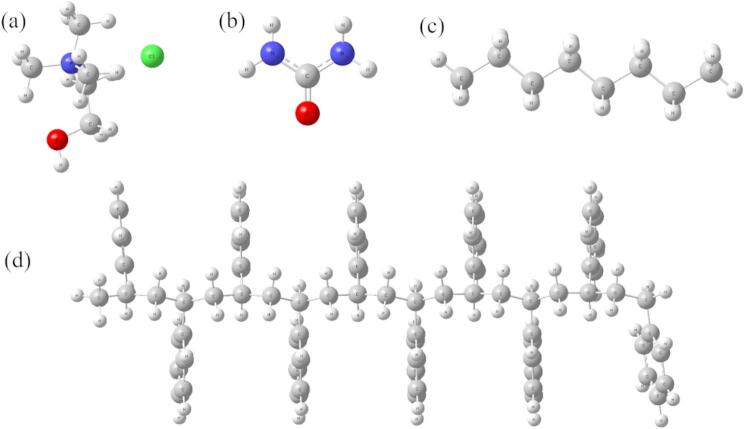
Chemical structures of (**a**) ChCl, (**b**) urea, (**c**) octane and (**d**) PS. Color key: grey (carbon); white (hydrogen); blue (nitrogen); red (oxygen); green (chloride).

**Fig. 2:**
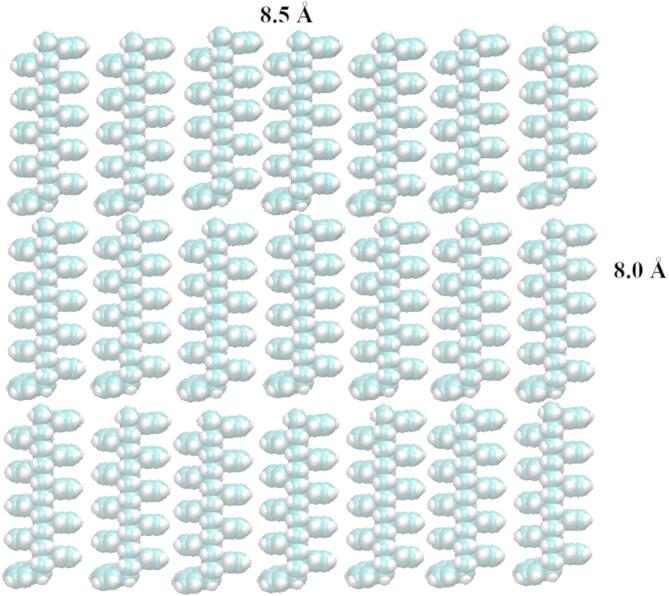
3D structure of the PS slab. The VDW representation uses grey for carbon atoms and white for hydrogen atoms.

PS was modeled as a surface formed by stacking 21 oligomeric chains, each consisting of ten repeating styrene units (Fig. 1, and 2). This configuration provided a chemically realistic and physically stable representation of the polymeric surface.

Figure 2 presents the three-dimensional structure of the PS slab, which was constructed using GaussView software^29^. The resulting model dimensions were approximately 8.5 Å × 8.5 Å.

The oil phase was modeled using a hydrocarbon compound, such as octane, which served as a representative non-polar organic liquid. The aqueous phase was modeled using the SPC/E water model, widely used for its accurate representation of water behavior in molecular simulations. The DES selected for this study was based on a 1:2 molar ratio of choline chloride (ChCl) and urea, a well-known combination used in numerous studies for its amphiphilic and interfacial tuning properties.

To balance computational cost and time, a limited number of octane molecules and a flat PS surface were selected for the model systems. Although these systems are simplified compared to real-world conditions (e.g., polydisperse oil droplets and rough membrane surfaces), they are sufficient to provide mechanistic insights at the atomistic scale. A similar modeling strategy has been adopted in earlier classical all-atom MD simulations on oil-water separation, where a small number of octane molecules were used to study interfacial phenomena^30–32^. Our future studies will expand the system size (united all atom MD simulations or coarse-grained MD simulations) and surface complexity to approach experimental realism more closely.

**Table 2:** Summarizes designed systems for classical all-atom MD simulations.

	PS	Octane	ChCl	Urea	Water
1	21	-	-	-	25,000
2	21	1	-	-	25,000
3	21	3	-	-	25,000
4	21	-	1	2	25,000
5	21	1	1	2	25,000
6	21	3	1	2	25,000

Table 2 presents the composition of six classical all-atom MD simulation systems designed to investigate the influence of DES components, ChCl and urea, on the behavior of a PS oligomer in an octane-based environment. Each system includes 21 PS molecules and a consistent amount of water (25000 molecules) to maintain hydration across all configurations. System 1 serves as the control, containing only PS and water. Systems 2 and 3 introduce 1 and 3 octane molecules, respectively, to examine the impact of increasing octane concentration on its intermolecular interactions with PS. System 4 introduces a DES component by including 1 ChCl and 2 urea molecules, providing insight into the role of DES alone. System 5 combines 1 ChCl and 2 urea molecules with a single octane molecule, simulating an oil-DES-PS-water environment. Finally, System 6 includes higher concentrations of octane components, namely, 3 octane molecules, allowing for evaluation of oil-DES-PS-water system in detail. This systematic variation enables a comparative understanding of how DES composition influences molecular interactions, oil-DES-PS-water behavior.

### Classical all-atom MD simulations

All molecular structures and topologies were prepared using the Automated Topology Builder (ATB) platform^33^ to ensure compatibility with the GROMOS54a7 force field^34^, which was employed throughout the simulations. The systems were first subjected to energy minimization using the steepest descent method to eliminate unfavorable steric clashes and relax atomic positions.

This was followed by equilibration in two stages: a 0.1 ns NVT (constant number of particles, volume, and temperature) equilibration at 298 K, and a subsequent 0.1 ns NPT (constant number of particles, pressure, and temperature) equilibration at 298 K and 1 bar to ensure stable density and pressure. Temperature control was achieved using the Nosé–Hoover thermostat with a coupling constant of 0.5 ps and a reference temperature of 298.15 K, applied to the entire system. Pressure control during the NPT equilibration was maintained using the Berendsen barostat with isotropic pressure coupling, a coupling constant of 4.0 ps, a reference pressure of 1 bar, and a compressibility of 4.5 × 10⁻⁵ bar⁻¹. The production classical all-atom MD simulations were then carried out for 30 ns under the NVT ensemble at 298 K using periodic boundary conditions in all three spatial dimensions to mimic bulk-phase behavior^35–37^. During the simulations, the PS surfaces were subjected to frozen constraints in the X, Y, and Z directions to maintain structural stability.

The long-range electrostatic interactions were treated using the Particle Mesh Ewald (PME) method with a grid spacing of 0.16 nm and a real-space cutoff of 1.0 nm for both Coulombic and van der Waals interactions^35–37^. Bond lengths were constrained using the LiNCS algorithm to enable a 2-femtosecond time step while preserving molecular geometry. After simulation, all trajectories were subjected to detailed analysis to quantify the interfacial properties and molecular interactions. Properties such molecular structures, radial distribution functions (RDFs), and interaction energies were evaluated to understand the role of DES at the oil–water interface. Visualization of molecular configurations and interface structures was carried out using Visual Molecular Dynamics (VMD), and further data analysis was performed using tools integrated into the GROMACS 2021.4 package^38,39^. The reliability of the applied GROMOS54a7 force field was verified by computing the density (0.98 g/cm³) and comparing it with the reported literature value for water density (1.0 g/cm³), as our system was solvated to reach unity, showing consistent behavior^40,41^. Although we did not explicitly calculate solvation free energies for validation in this work, we previously performed related computations using the GROMOS54a7 force field for an anion exchange membrane system, where the calculated free energies closely matched reference values, further supporting the validity of this force field^42^.

This computational approach enables a molecular-level understanding of how DES components organize at the interface and interact with both the PS surface and the surrounding oil and water phases. The insights gained from these simulations contribute to the broader goal of designing more effective and sustainable separation systems for oil–water mixtures using functionalized solvents and polymeric supports.

## Results and discussion

To explore the role of DES in modulating interfacial behavior, the molecular structure and interactions of the PS surface in the presence of water and oil phases were systematically analyzed. This section presents a comparative discussion of the intermolecular interactions occurring in two distinct environments: (i) water only, and (ii) a biphasic system consisting of water and octane. The results are divided into two main parts, each detailing the molecular arrangement and radial distribution profiles of key system components. The insights gained provide a molecular-level understanding of how DES components interact with PS under varying solvation conditions.

### PS supported by a DES system in water

#### Molecular structural analysis

**Fig. 3:**
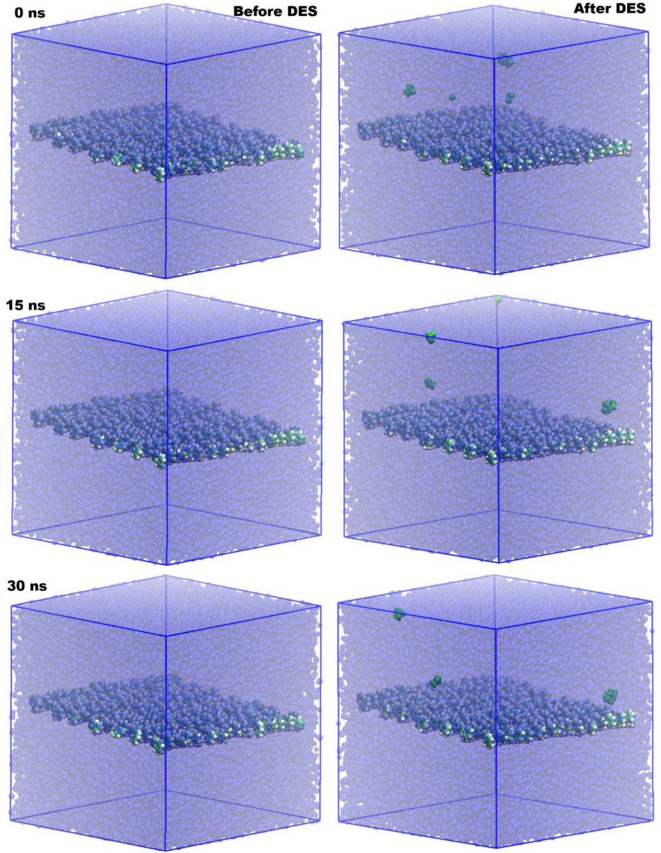
Molecular structures of the PS-based membrane in the presence and absence of DES in water. Color scheme: transparent blue represents water molecules; PS is shown in standard VDW coloring at the center; green denotes DES molecules.

Figure 3 provides compelling visual evidence of the dynamic interplay between PS, water, and DES molecules over time, offering critical insights into the structural evolution of the system.

The snapshots at 0 ns, 15 ns, and 30 ns, both before and after the introduction of DES, illustrate significant changes in the molecular arrangement and distribution. In all panels, the transparent blue represents water molecules, the central layer in standard VDW coloring denotes PS, and the green spheres highlight the DES molecules.

Before the introduction of DES (left column), the PS membrane remains largely intact and relatively stable within the aqueous environment across all time points (0 ns, 15 ns, and 30 ns). The water molecules (transparent blue) are homogeneously distributed around the PS layer, indicating a conventional interface where water interacts directly with the polymer surface. This initial state serves as a crucial baseline for understanding the profound effects induced by the DES.

Upon the introduction of DES (right column), the molecular structures undergo a noticeable transformation. At 0 ns “After DES,” the green DES molecules are initially distributed somewhat randomly, with some already beginning to interact with the PS surface while others are dispersed within the water phase. As time progresses to 15 ns “After DES,” a clear trend emerges: the DES molecules (green) begin to preferentially accumulate around and on the PS surface. This accumulation is not merely superficial; some DES molecules appear to be penetrating or adsorbing onto the PS layer. This observation suggests a strong affinity between the DES and PS, which likely alters the interfacial properties. Furthermore, a scattering of DES molecules away from the main PS surface is also observed, indicating some solubility or dispersion of DES within the bulk water phase, albeit with a preference for the PS interface.

By 30 ns “After DES,” the concentration of DES molecules at the PS interface appears to intensify, forming a more distinct layer or localized presence on the polymer surface. While a few DES molecules are still seen dispersed in the water phase, the overwhelming majority are associated with the PS. This sustained and enhanced interaction over time strongly suggests that the DES acts as an interfacial modifier, potentially altering the hydrophobicity or surface energy of the PS. This selective partitioning and accumulation of DES at the PS-water interface are crucial for understanding its role in mediating oil-water interfacial behavior, as it implies a direct interaction that could facilitate or stabilize oil-PS interactions in an aqueous environment. The migration and localization of DES molecules around the PS effectively create a modified surface, which is hypothesized to be key in altering the overall interfacial phenomena.

#### RDF analysis

**Fig. 4:**
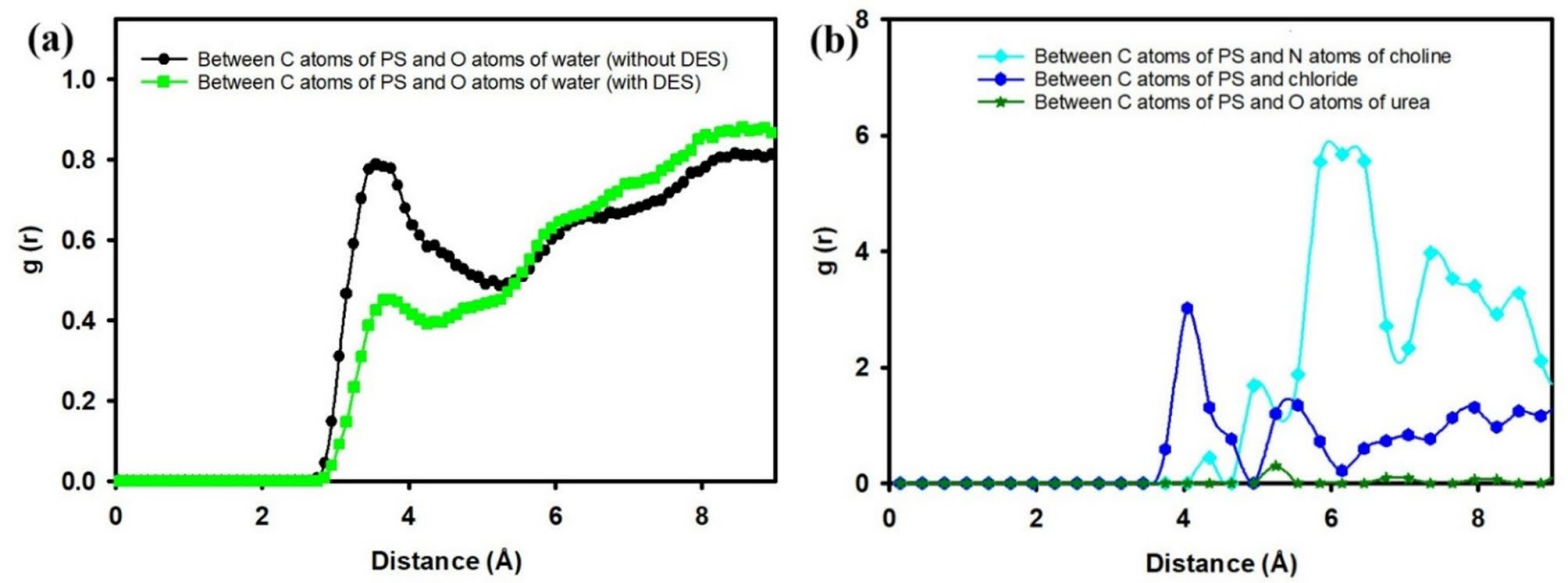
RDF analysis: (**a**) between carbon atoms of PS and oxygen atoms of water; (**b**) between carbon atoms of PS and DES components, both in the absence and presence of DES in water.

Figure 4 presents the RDFs, g(r), offering quantitative insights into the local molecular ordering and interactions within the system, both in the presence and absence of DES. Specifically, Fig. 4(a) illustrates the RDF between carbon atoms of PS and oxygen atoms of water, while Fig. 4(b) details the interactions between carbon atoms of PS and the individual components of the DES (nitrogen atoms of choline, chloride ions, and oxygen atoms of urea).

The RDFs in Fig. 4(a) reveal significant changes in the hydration shell around the PS surface upon the addition of DES. In the absence of DES (black line), the first prominent peak for the interaction between PS carbon atoms and water oxygen atoms is observed at approximately 3.7 A˚, with a peak value of about 0.8. This peak signifies the presence of a well-defined hydration layer directly adjacent to the PS surface, indicating strong immediate interactions between the hydrophobic PS and water molecules. The subsequent trough and gradual rise suggest a less ordered arrangement of water molecules further away from the surface.

In contrast, with the presence of DES (green line), the first peak for the PS carbon-water oxygen interaction shifts slightly to a shorter distance, around 3.5 A˚, and its intensity is notably reduced, reaching a peak value of approximately 0.45. This reduction in peak height and slight shift indicates a disruption of the direct PS-water interactions. The lower intensity suggests that fewer water molecules are in direct contact with the PS surface, implying that the DES molecules are preferentially occupying these interfacial sites, effectively displacing water. This observation strongly supports the hypothesis that DES modifies the hydration layer of PS, making the surface less accessible to water.

Figure 4(b) elucidates the specific interactions between the PS carbon atoms and the individual components of the DES: choline (via nitrogen atoms), chloride ions, and urea (via oxygen atoms). The most striking feature is the prominent first peak for the interaction between PS carbon atoms and nitrogen atoms of choline (cyan line), occurring at approximately 6.2 A˚ with a remarkably high peak value of around 5.7. This strong and sharp peak indicates a very close and highly ordered association between the choline cation of the DES and the PS surface. This suggests that choline plays a dominant role in the interfacial adsorption of DES onto PS.

The interaction between PS carbon atoms and chloride ions (blue line) also shows a distinct peak, albeit less intense than that of choline, appearing at approximately 4.0 A˚ with a peak value of about 3.0. This indicates that chloride ions also interact directly with the PS surface, possibly due to electrostatic interactions or specific binding sites. The closer proximity of chloride ions compared to choline, as suggested by the peak position, might point to different modes of interaction.

Conversely, the RDF between PS carbon atoms and oxygen atoms of urea (green line) shows a much weaker and broader interaction, with a very low peak intensity (less than 0.5) and a broad distribution starting around 4.5 A˚. This suggests that urea molecules have a significantly weaker or more diffuse interaction with the PS surface compared to both choline and chloride. The observed trends in Fig. 4(b) collectively demonstrate that the DES components exhibit differential affinities for the PS surface, with choline showing the strongest and most direct interaction, followed by chloride ions, while urea contributes minimally to direct surface binding.

### Intermolecular interactions of PS supported by a DES system in water and octane

#### Molecular structural analysis

**Fig. 5:**
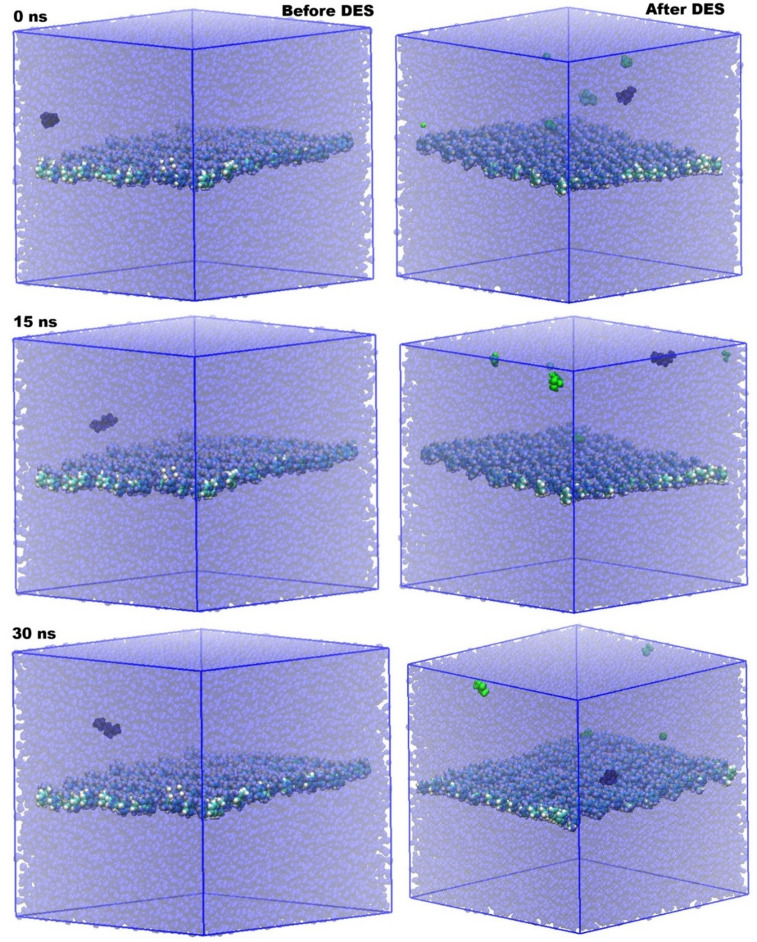
Molecular structures of the PS-based membrane in the presence and absence of DES in water and octane. Color scheme: transparent blue represents water molecules; PS is shown in standard VDW coloring at the center; green denotes DES molecules; black denotes octane molecule.

Figure 5 provides a comprehensive visual representation of the molecular structures of the PS-based membrane in the presence and absence of a DES within a mixed water and octane environment. The color scheme is consistent: transparent blue for water, standard VDW coloring for PS at the center, green for DES molecules, and black for octane molecules. These snapshots, taken at 0 ns, 15 ns, and 30 ns, offer crucial insights into the dynamic interactions and distribution of all components, particularly highlighting the role of DES in mediating the PS-oil-water interface.

In the “Before DES” scenario (left column), the system primarily consists of PS, water, and octane. At 0 ns, the octane molecules (black) are initially positioned above the PS layer, separated from it by water. As the simulation progresses to 15 ns and 30 ns, the octane molecules show a tendency to remain in the upper aqueous phase, with limited direct interaction or penetration into the PS layer. The water molecules (transparent blue) surround the PS, forming a distinct interface. This observation suggests that, in the absence of DES, the PS surface maintains its inherent characteristics, and the octane, being immiscible with water and having a relatively weak affinity for PS, tends to stay separated from the polymer by the aqueous phase. There is no clear indication of octane spreading or adhering to the PS surface, reinforcing the barrier effect of the water layer.

The “After DES” scenario (right column) presents a dramatically different Picture. At 0 ns, the DES molecules (green) are introduced into the system, initially dispersed within the water phase and near the PS surface, alongside the octane molecules (black). As time evolves to 15 ns, a notable phenomenon occurs: the DES molecules begin to migrate towards and adsorb onto the PS surface. Crucially, the octane molecules (black) also show a clear tendency to move closer to the PS surface, often appearing in proximity to the adsorbed DES molecules. This co-localization suggests a synergistic effect where DES facilitates the interaction between octane and PS.

By 30 ns “After DES,” the accumulation of both DES and octane molecules at the PS interface becomes even more pronounced. The DES molecules appear to form an interfacial layer on the PS, and the octane molecules are observed to be in much closer contact with the PS surface than in the “Before DES” case. The presence of DES seems to alter the surface properties of PS, making it more amenable to interaction with the oil phase. This visual evidence strongly supports the hypothesis that DES plays a pivotal role in modifying the PS-water-octane interface, leading to enhanced oil-PS interactions, which is fundamental to understanding oil-water interfacial behavior in these systems.

#### RDF analysis

**Fig. 6:**
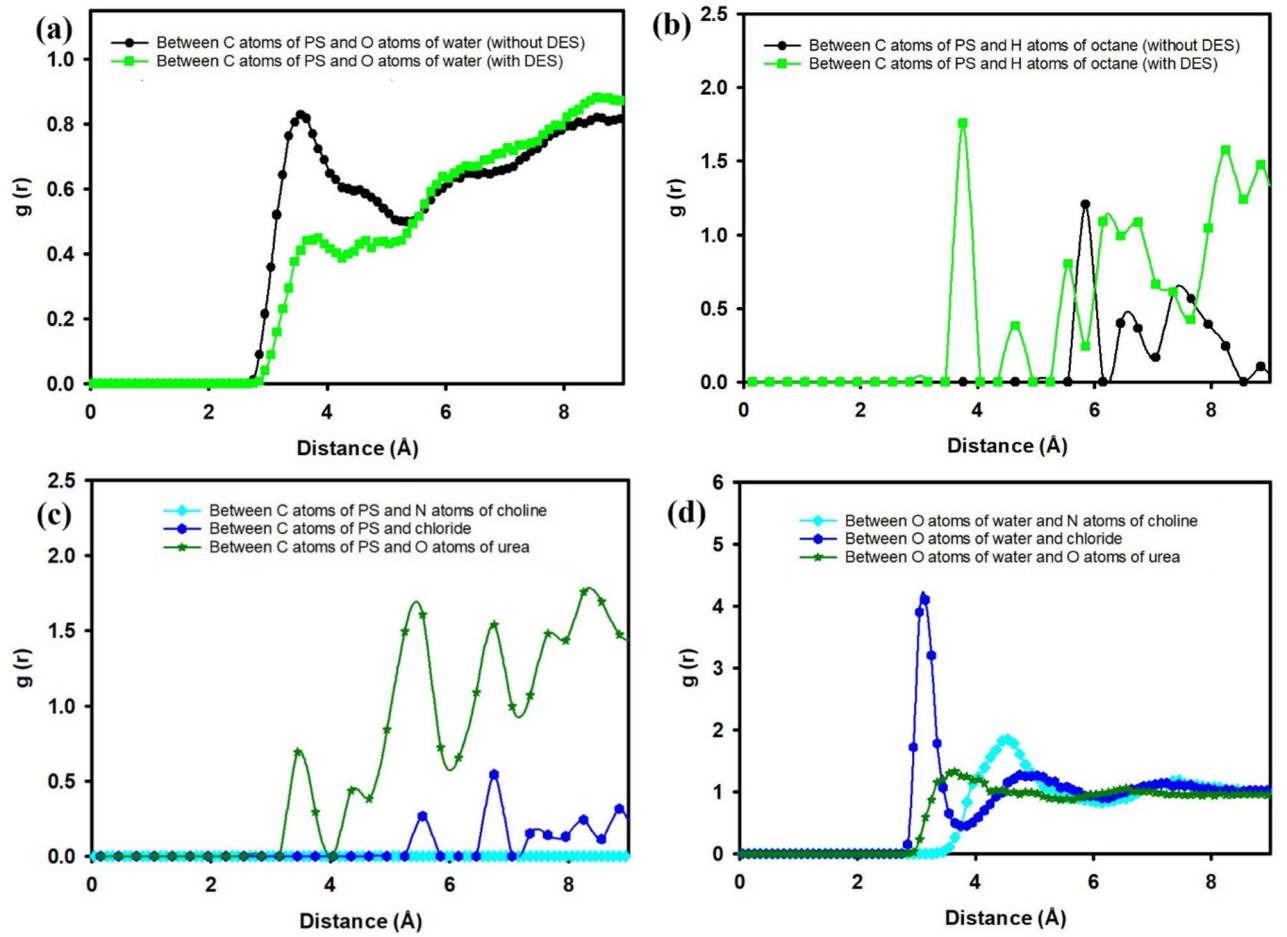
RDF analysis: (**a**) between carbon atoms of PS and oxygen atoms of water; (**b**) between carbon atoms of PS and hydrogen atoms of octane; (**c**) between carbon atoms of PS and DES components; (**d**) between oxygen atoms of water and DES components, both in the absence and presence of DES in water and octane.

Figure 6 presents a comprehensive RDF analysis, elucidating the intricate intermolecular interactions within the PS-DES-water-octane system. This figure is segmented into four distinct panels, each offering unique insights into the spatial correlation between different molecular species.

Figure 6(a) illustrates the RDF between the carbon atoms of PS and the oxygen atoms of water. In the absence of DES (black line), a distinct first peak is observed at approximately 3.7 A˚ with a peak value of about 0.8. This indicates a well-defined hydration shell around the PS surface. Upon the addition of DES (green line), this first peak shifts slightly to a closer distance, around 3.5 A˚, and its intensity is notably reduced to about 0.45. This decrease in peak height and slight shift signifies a reduction in direct PS-water interactions, suggesting that DES molecules preferentially occupy the interfacial region, displacing water molecules from the immediate vicinity of the polystyrene surface.

Figure 6(b) illustrates the RDF between the carbon atoms of PS and the hydrogen atoms of octane, comparing the cases without and with DES. In the absence of DES (black line), a broad and less intense first peak appears at around 6.0 Å, with additional weaker oscillations beyond 7 Å. This indicates relatively weak and diffuse octane–PS interactions, reflecting limited affinity of octane molecules for the hydrophobic polystyrene surface under these conditions. However, upon the addition of DES (green line), the first significant peak shifts closer to around 4.0 Å, with a much sharper and higher intensity (≈ 2.1), followed by stronger oscillatory patterns extending to larger distances. The pronounced increase in peak height and closer positioning suggest that DES facilitates stronger and more structured interactions between octane and PS. This behavior supports the hypothesis that DES molecules enable us to enhance octane adsorption and spreading on the PS surface.

Figure 6(c) focuses on the direct interactions between PS carbon atoms and the DES components. The interaction with the nitrogen atoms of choline (cyan line) shows a prominent peak around 6.2 A˚ with a g(r) value of approximately 1.6. This strong peak confirms a significant and ordered interaction between choline and the PS surface, indicating that choline plays a primary role in bridging the gap between PS and other components. The interaction with chloride ions (blue line) presents a broader and less intense peak around 4.0 A˚ with a g(r) value of about 0.3, suggesting a weaker yet present association. Notably, the interaction with oxygen atoms of urea (green line) is almost negligible, indicating that urea has minimal direct interaction with the PS surface in this multicomponent system.

Figure 6(d) examines the interactions between water oxygen atoms and the DES components. A very strong and sharp peak is observed between water oxygen and chloride ions (blue line) at approximately 3.2 A˚, with a high g(r) value exceeding 4.0. This signifies a highly organized first hydration shell around chloride ions, indicative of strong hydrogen bonding or electrostatic interactions. Interactions between water oxygen and choline nitrogen (cyan line) show a broader peak around 4.5 A˚ with a g(r) value of about 1.5, suggesting a less structured but still significant hydration of choline. Finally, the interaction between water oxygen and urea oxygen (green line) presents a peak around 3.0 A˚ with a g(r) value of about 1.2, consistent with hydrogen bonding between water and urea. These results collectively highlight the differential hydration of DES components, which in turn influences their partitioning and interaction with the PS surface and the overall interfacial behavior.

#### Interaction energy analysis

Table 3 provides a quantitative assessment of the interaction energies (electrostatic and van der Waals, vdW) between PS and various components, octane, choline, chloride, and urea, both in the absence and presence of DES. These energy values are crucial for understanding the driving forces behind the molecular arrangements observed in Fig. 5 and the radial distribution functions in Fig. 6.

**Table 3:** Interaction energies for the polystyrene-based membrane in the presence and absence of DES in water and octane.

Interaction energies between (kJ/mol)			PS and octane	PS and choline	PS and chloride	PS and urea
Without DES	1 octane	Electrostatic	0.15	-	-	-
		vdW	−10.05	-	-	-
	3 octanes	Electrostatic	0.55	-	-	-
		vdW	−36.46	-	-	-
With DES	1 octane	Electrostatic	0.44	−0.25	−0.041	−0.59
		vdW	−23.88	−3.52	−0.021	−4.11
	3 octanes	Electrostatic	0.38	−6.38	−0.14	−0.76
		vdW	−22.37	−16.59	−0.061	−4.71

In the absence of DES, the interaction between PS and octane is dominated by van der Waals forces. For a single octane molecule, the vdW interaction energy with PS is − 10.05 kJ/mol, while the electrostatic component is a negligible 0.15 kJ/mol. When three octane molecules are present, the vdW attraction increases significantly to − 36.46 kJ/mol, consistent with increased contact area and cumulative non-polar interactions. The electrostatic contribution remains minimal (0.55 kJ/mol). These values indicate a weak but attractive interaction between PS and octane in a purely aqueous environment, primarily driven by hydrophobic forces.

The introduction of DES dramatically alters the PS-octane interaction. For a single octane molecule, the vdW attraction with PS strengthens to − 23.88 kJ/mol in the presence of DES, a significant increase compared to the − 10.05 kJ/mol without DES. This enhancement suggests that DES facilitates a more favorable interaction between PS and octane. Interestingly, for three octane molecules, the vdW interaction is − 22.37 kJ/mol, which is less negative than the − 36.46 kJ/mol observed without DES. This seemingly counterintuitive result might indicate that while DES promotes initial PS-octane contact, it could also compete for surface sites or alter the octane’s conformation, leading to a slightly less favorable cumulative vdW interaction for multiple octane molecules compared to a scenario where water is the only intervening medium and octane can fully collapse onto the surface.

This seemingly counterintuitive result might indicate that while DES promotes initial PS–octane contact, it could also compete for surface sites or alter the orientation of octane molecules, leading to a slightly less favorable cumulative vdW interaction for multiple octane molecules compared to a scenario where water is the only intervening medium and octane can fully collapse onto the surface. In other words, the presence of DES introduces competing intermolecular interactions (e.g., between PS–DES and DES–octane) that partially offset the direct PS–octane vdW attraction, explaining why the cumulative energy for multiple octanes appears less favorable despite enhanced interfacial compatibility. Moreover, this interpretation is consistent with the RDF results in Fig. 6(b), where for a single octane molecule, DES induces a sharper and closer first peak for PS–octane interactions, confirming stronger local adsorption.

In the presence of DES, the interactions between PS and the DES components are also critical. Choline shows a substantial vdW interaction with PS, with values of − 3.52 kJ/mol for 1 octane and − 16.59 kJ/mol for 3 octanes. The electrostatic interaction with choline is also notable, particularly with 3 octanes (− 6.38 kJ/mol). This strong attraction, especially the vdW component, confirms the high affinity of choline for the polystyrene surface, consistent with the prominent peak observed in the RDF analysis (Fig. 5c).

Conversely, chloride ions exhibit very weak interactions with PS, with vdW energies of only − 0.021 kJ/mol (1 octane) and − 0.061 kJ/mol (3 octanes), and negligible electrostatic contributions. This indicates that chloride ions do not directly contribute significantly to the binding with the polystyrene surface. Urea also shows relatively weak vdW interactions (− 4.11 kJ/mol for 1 octane, − 4.71 kJ/mol for 3 octanes) and minor electrostatic contributions (− 0.59 kJ/mol and − 0.76 kJ/mol respectively). However, despite these weak direct interactions (RDF analysis, interaction energy analysis), urea still plays an indirect yet important role in stabilizing the DES structure through extensive intermolecular interactions with choline and chloride, thereby maintaining the integrity of the DES network at the interface. This network stabilization may indirectly influence the overall compatibility of PS with oil and water, highlighting that urea’s contribution is more structural than surface-specific.

These energy analyses confirm that DES, particularly its choline component, plays a crucial role in mediating the PS-octane interface. The enhanced vdW attraction between PS and octane in the presence of DES, coupled with the strong affinity of choline for PS, suggests that DES acts as an interfacial bridge, promoting the interaction between the hydrophobic polymer and the oil phase. The differential interactions of DES components with PS highlight the specific roles of choline in modifying the polystyrene surface properties.

## Conclusion

In this study, we employed classical all-atom molecular dynamics simulations to thoroughly investigate the molecular-level interactions at the oil-water-deep eutectic solvent-polystyrene interface, aiming to unlock new insights into interfacial behavior and separation performance. The findings provide a comprehensive understanding of how DES modifies the polystyrene surface and its interactions within a complex multiphase system.

Our molecular structural analysis distinctly demonstrated the transformative role of DES. In the absence of DES, the polystyrene-water interface remained relatively stable, with octane maintaining a separated phase. However, upon DES introduction, DES molecules rapidly migrated to and adsorbed onto the polystyrene surface, acting as an interfacial bridge that significantly enhanced the co-localization and direct contact of octane molecules with the polymer.

The RDF analysis provided quantitative evidence for these structural changes. We observed a notable reduction in the direct interactions between polystyrene and water in the presence of DES, signified by a decrease in the first peak of the PS carbon-water oxygen RDF. This indicates that DES effectively displaces water from the immediate vicinity of the PS surface. Crucially, the RDFs also highlighted a strong and specific interaction between PS carbon atoms and the nitrogen atoms of choline, a key component of the DES. In contrast, chloride and urea showed much weaker direct interactions with the polystyrene surface.

The interaction energy analysis further corroborated these observations, revealing the energetic favorability of DES-mediated interactions. Specifically, the van der Waals attraction between polystyrene and octane was significantly enhanced when DES was present, particularly for single octane molecules, transitioning from − 10.05 kJ/mol without DES to − 23.88 kJ/mol with DES. This enhancement is primarily driven by the strong vdW and electrostatic interactions between polystyrene and choline, which effectively reduces the interfacial energy between the hydrophobic PS and octane.

While the present models are simplified, the findings provide critical atomistic insights into the interactions of octane with PS and the role of DES components in modulating these interfaces. Future studies will extend these simulations to larger, more realistic systems (e.g., higher octane loading and non-flat PS surfaces, coarse-grained MD simulations) to further bridge the gap between computational study and experimental conditions.

In conclusion, this computational study provides fundamental molecular-level insights into how deep eutectic solvents modulate interfacial behavior on polystyrene surfaces. By promoting favorable interactions between polystyrene and octane while concurrently influencing the water-polystyrene interface, DES demonstrates significant potential as an environmentally friendly additive for enhancing the performance of polymer-based membranes in oil-water separation applications. These findings offer a robust molecular basis for the rational design and development of next-generation DES-assisted membrane systems for efficient and sustainable oil-water separation.

## Data Availability

The datasets generated during and/or analyzed during the current study are available from the corresponding author on reasonable request.

## Abbreviations

CA: Cellulose acetate
ChCl: Choline chloride
DES: Deep eutectic solvent
DMSO: Dimethyl sulfoxide
MD: Molecular dynamics
RDFs: Radial distribution functions
PVDF: Polyvinylidene fluoride
PEGDA: Poly polyethylene glycol diacrylate
PSF: Poly sulfone
PS: Poly styrene
